# Utilization of automated cilia analysis to characterize novel *INPP5E* variants in patients with non-syndromic retinitis pigmentosa

**DOI:** 10.1038/s41431-024-01627-6

**Published:** 2024-05-28

**Authors:** Kae R. Whiting, Lonneke Haer-Wigman, Ralph J. Florijn, Ronald van Beek, Machteld M. Oud, Astrid S. Plomp, Camiel J. F. Boon, Hester Y. Kroes, Ronald Roepman

**Affiliations:** 1 Department of Human Genetics, Radboud University Medical Center, Nijmegen, The Netherlands; 2 Department of Human Genetics, Amsterdam UMC, University of Amsterdam, Amsterdam, The Netherlands; 3 Department of Ophthalmology, Amsterdam UMC, University of Amsterdam, Amsterdam, The Netherlands; 4 Department of Ophthalmology, Leiden University Medical Center, Leiden, The Netherlands; 5 Department of Medical Genetics, University Medical Center Utrecht, Utrecht, The Netherlands

**Keywords:** Rare variants, Diseases

## Abstract

*INPP5E* encodes inositol polyphosphate-5-phosphatase E, an enzyme involved in regulating the phosphatidylinositol (PIP) makeup of the primary cilium membrane. Pathogenic variants in *INPP5E* hence cause a variety of ciliopathies: genetic disorders caused by dysfunctional cilia. While the majority of these disorders are syndromic, such as the neuronal ciliopathy Joubert syndrome, in some cases patients will present with an isolated phenotype—most commonly non-syndromic retinitis pigmentosa (RP). Here, we report two novel variants in *INPP5E* identified in two patients with non-syndromic RP: patient 1 with compound heterozygous variants (c.1516C > T, p.(Q506*), and c.847G > A, p.(A283T)) and patient 2 with a homozygous variant (c.1073C > T, p.(P358L)). To determine whether these variants were causative for the phenotype in the patients, automated ciliary phenotyping of patient-derived dermal fibroblasts was performed for percent ciliation, cilium length, retrograde IFT trafficking, and INPP5E localization. In both patients, a decrease in ciliary length and loss of INPP5E localization in the primary cilia were seen. With these molecular findings, we can confirm functionally that the novel variants in *INPP5E* are causative for the RP phenotypes seen in both patients. Additionally, this study demonstrates the usefulness of utilizing ciliary phenotyping as an assistant in ciliopathy diagnosis and phenotyping.

## Introduction

Inherited retinal disorders (IRDs) are genetically and phenotypically heterogeneous disorders caused by genetic defects in ~280 genes that lead to the progressive degeneration of photoreceptors in the retina [1–3]. The most common IRD is retinitis pigmentosa (RP, OMIM#268000), which is known to affect every 1:4000 people worldwide [4–6]. RP can present as an isolated phenotype, known as non-syndromic RP, or as one clinical feature in a disorder that affects multiple organs and tissues, such as ciliopathies. Ciliopathies can involve defects in several organs including the kidney, liver, and central nervous system alongside retinal degeneration [1, 2, 7–9].

Ciliopathies occur when a pathogenic variant leads to the disruption to the structure or function of the primary cilium, a small microtubule-based organelle that extends from the apical surface of most post-mitotic cells. Primary cilia play a vital role in the regulation of organismal development and tissue homeostasis through their function in cellular signaling [10–13] and cell cycle regulation [14, 15]. Moreover, specialized sensory cilia are present throughout the body that are modified for specific functions, for example, the photoreceptor in the retina, which is the primary source for light capture and transduction in many organisms [16]. The main compartment of the photoreceptor is the outer segment (OS), a highly modified axoneme containing hundreds of opsin-filled discs that allow for phototransduction to occur [16, 17]. The OS is connected to the rest of the photoreceptor via an elongated transition zone, known as the connecting cilium (CC), which allows for the trafficking of specific proteins in and out of the OS [17]. Variants in OS and CC proteins can lead to RP; and as many photoreceptor proteins are also found in the primary cilium, retinopathies are frequently observed as a clinical phenotype in ciliopathies [18]. One such ciliopathy with a high prevalence of RP is Joubert Syndrome (JS, OMIM #PS213300). JS is characterized primarily by a malformation in the mid-hindbrain known as the Molar Tooth Sign (MTS), which is identifiable by magnetic resonance imaging of the brain [19]. JS patients present with a variety of phenotypes, including intellectual disability (ID), retinal degeneration, skeletal abnormalities, and renal anomalies, among several other clinical features [20, 21]. Pathogenic variants in at least 30 genes have been implicated in causing JS [22] including *Inositol Polyphosphate-5-Phosphatase E (INPP5E*, NM_019892.6).

*INPP5E* encodes a 72 kDa phosphatase that is localized in the axoneme of the primary cilium and plays an important role in ciliary regulation of the PI3K signaling pathway. INPP5E’s primary function is to cleave the 5′ phosphate group from phosphatidylinositol 4,5-bisphosphate (PI(4,5)P_2_) or from phosphatidylinositol (3,4,5)-trisphosphate (PI(3,4,5)_3_) to form phosphatidylinositol 4-phosphate (PI4P) or phosphatidylinositol 3,4-bisphosphate (PI(3,4)_2_), respectively [14, 23, 24]. Through its enzymatic activity in the axoneme of the cilium, INPP5E plays a key role in regulating the phospholipid makeup of the ciliary membrane, giving the cilium a unique lipid composition compared to the remainder of the cell. Dynamic modulation of the specific lipids present in the ciliary membrane has been found to regulate ciliogenesis [14], cilia stability [25], and ciliary signaling [23, 24]. Moreover, INPP5E has been identified as a regulator of autophagy [26, 27] and interacts with several intraflagellar transport (IFT) proteins [28]. Within the retina, INPP5E is localized in the inner segment and CC of the photoreceptor [29], however the precise function within the photoreceptor is still poorly understood.

In this study we report two patients with non-syndromic RP with novel variants in *INPP5E* of initially unknown significance: Patient 1 is a 17-year-old female with compound heterozygous variants (c.1516C > T, p.(Q506*), and c.847G > A, p.(A283T)) and Patient 2 is a 57-year-old male with a homozygous variant (c.1073C > T, p.(P358L)). Using dermal fibroblasts derived from both patients, ciliary phenotyping was performed using ALPACA (Accumulation and Length Phenotype Automated Cilia Analysis) [30] for percentage ciliation, cilium length, retrograde IFT trafficking, and INPP5E localization. We detected aberrant values for both the cilium length and INPP5E localization parameters, indicating that the identified variants are likely causative for the RP phenotypes in both patients.

## Methods

### Patient genotyping

For patient 1, next-generation sequencing of the genes (including the INPP5E gene) in the vision panel and variant classification of variants in the analyzed genes was performed similarly as described previously [31]. All coding exons of the genes, including the 20 flanking intron nucleotides were analyzed. Target enrichment was done with custom designed in solution captures (SeqCap EZ Choice, Nimblegen) using the Nimblegen Rebal algorithm. Samples on the vision panels were all sequenced on the MiSeq. MiSeq paired-end sequencing reads (2 × 150 bp) were mapped to GRCh37/hg19 reference genome using BWA-MEM (0.7.5). Variants were identified using the HaplotypeCaller from GATK version 2.8.1 (Genome Analysis Toolkit, Broad Institute) along with Picard tools version 1.89. Sample swap was ruled out by using an SNP check on an independent DNA dilution (genotyping at least 10 frequently occurring SNPs).

For patient 2, exome sequencing and data analysis were performed according to routine diagnostic procedures [32], in the ISO15189 accredited Genome Diagnostic Laboratory of the Radboud University Medical Center (Nijmegen, the Netherlands). In short, next-generation sequencing was performed on a DNBseq at BGI-Europa after enrichment with the Agilent SureSelectQXT Human All Exon v5 Kit. Read alignment was performed using BWA and variant calling using GATK and Conifer. Variants present in genes associated with visual impairment (vision disorders gene panel version DG-2.17 consisting of 432 genes (available at https://www.radboudumc.nl/getmedia/d1c8d115-ae1e-4d0a-8f49-307c1134a49c/VISIONDISORDERS_DG217.aspx) were interpreted. All copy number variants containing a gene from the vision disorder gene panel were interpreted and classified. Moreover, nucleotide variants present in genes from the vision disorder gene panel that were classified as pathogenic by HGMD and nucleotide variants present in genes from the vision disorder gene panel with a frequency below 5% in the dbSNP database and below 1% in an in-house database (consisting of exome sequencing data of 24,488 individuals mainly of Dutch origin) and within the exon or within the intronic position of −8 to +3 were interpreted and classified based on existing variant classification guidelines established by the Association for Clinical Genetic Science.

### Fibroblast culture and immunofluorescent staining

Skin-derived fibroblasts were cultured in Dulbecco’s modified Eagle’s medium (DMEM) with 20% fetal bovine serum (FBS), 1% sodium pyruvate and 1% penicillin-streptomycin. Cells were grown on 18 mm Ø glass coverslips and grown until 80% confluency for immunofluorescent analysis. To stimulate cilia formation, cells were serum starved using serum starvation medium (DMEM with 0.2% FBS) 48 h prior to fixation. The cells were then fixed in 2% paraformaldehyde (PFA) in phosphate-buffered saline (PBS) for 20 min and permeabilized in 1% Triton-X-100 in PBS for 5 min. Cells were blocked for 20 min in 2% bovine serum albumin (BSA) in PBS, and incubated in primary antibodies diluted in 2% BSA for 1 h at room temperature or overnight at 4 °C. The cells were washed in PBS prior to incubation with secondary antibodies for 1 h at room temperature. The primary antibodies used were: anti-acetylated- α-tubulin (mouse monoclonal, T6793, Sigma-Aldrich, Saint Louis, USA, 1:1000), anti-ARL13B (Rabbit polyclonal, 17711-1-AP, Proteintech Group, Manchester, UK, 1:500), anti-Pericentrin (PCNT) (mouse monoclonal, AB28144, Abcam, Cambridge, UK, 1:1000), anti-IFT88 (rabbit polyclonal, 13967-1-AP, Proteintech Group, Manchester, UK, 1:500), and anti-INPP5E (rabbit polyclonal, 17797-1-AP, Proteintech Group, Manchester, UK, 1:500). Secondary antibodies used: anti-rabbit Alexa Fluor 488, anti-mouse IgG2b Alexa Fluor 568, anti-mouse IgG1 Alexa Fluor 647, and anti-mouse Alexa Fluor 568 (ThermoFisher Scientific, Waltham, USA, 1:500). Coverslips were embedded in Fluoromount-G with DAPI (Southern Biotech, Birmingham, AL, USA) on microscopic glass slides. Immunofluorescence of samples was performed using a Zeiss Axio Imager Z2 fluorescence microscope (Zeiss, Sliedrecht, Netherlands) equipped with ApoTome slider, using a 63× oil objective. Images were acquired and processed as described by Doornbos et al. [30], with five images taken per biological replicate and three biological replicates performed per analysis.

### Ciliary phenotype

Ciliary phenotyping was performed via the Accumulation and Length Phenotype Automated Cilia Analysis (ALPACA) tool [30]. Ciliation percentage, cilium length, and retrograde IFT trafficking were investigated for each healthy control cell line to determine a “healthy” cilium phenotype before using the same analysis for each patient line to determine how the variant affects the ciliary phenotype. Ciliation was calculated manually as the percent of DAPI-positive cells with extended axonemes, determined by staining for ARL13B and acetylated-α-tubulin. Cilium length was measured based on ciliary staining using ARL13B, acetylated-α-tubulin, and PCNT. Optimal thresholding of the images was determined prior to automated measuring via the ALPACA tool. To measure retrograde IFT trafficking in the cilia, accumulation of IFT88 along the cilium was calculated using parameters previously defined for use with ALPACA. Optimal thresholding was determined prior to automated analysis, using acetylated-α-tubulin and PCNT as ciliary markers alongside IFT88. Finally, INPP5E expression and localization were determined manually as the percent of acetylated-α-tubulin positive stained cilia with co-expression of INPP5E in the axoneme.

### Statistical analysis

Ciliation percentage was calculated per image while length, IFT accumulations, and INPP5E localization were calculated per cilium. Five images were taken per replicate, three replicates were performed per experiment, and one to two experiments were repeated per cell line. Statistical analysis was performed as One-way ANOVA with post-hoc Tukey’s Test using GraphPad Prism version 9.0.0 for Windows, GraphPad Software, San Diego, California USA www.graphpad.com. Data are represented as mean and standard deviation (SD).

## Results

### Clinical phenotype of patients

**Fig. 1:**
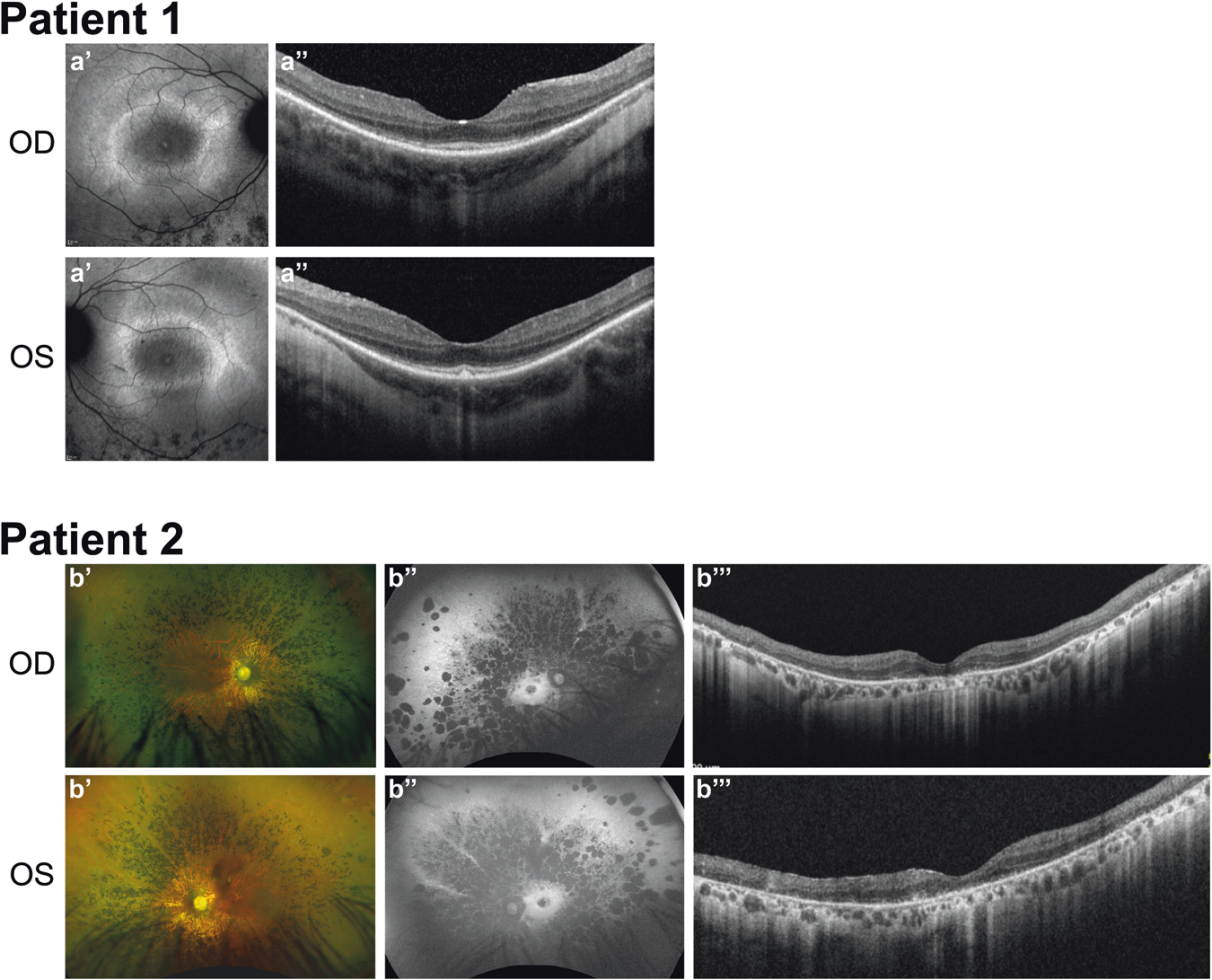
Clinical phenotypes of patients. Patient 1. **a’** Fundus autofluorescence images showing a typical hyperautofluorescent ring in the macula and hypo-autofluorescent spots outside the macula. **a”** Optical coherence tomography (OCT) images of the macula, showing typical thinning of the outer retinal layers (ellipsoid zone and external limiting membrane) outside the central macula 1. Patient 2. **b’** Wide-field fundus photograph showing extensive hyperpigmentations in the context of retinitis pigmentosa. **b”** Fundus autofluorescence showing a hyperautofluorescent ring in the macula, and extensive areas of hypo-autofluorescent atrophy of the retinal pigment epithelium. **b”’** OCT for showing extensive outer retinal atrophy.

In this study, two unrelated patients with a non-syndromic retinal phenotype were assessed. Patient 1 is a 17-year-old female who experienced blurry vision in the right eye since age 17, had difficulties reading, and had moderate night blindness. Her visual acuity was 20/30 in both eyes and visual field testing showed pericentral and mid-peripheral visual field loss. OCT of the macula showed thinning of the outer retinal layers (ellipsoid and external limiting membrane) (Fig. 1a). Fundus autofluorescence also showed a hyperautofluorescent ring in both eyes, typical of RP, with some hypo-autofluorescent zones in the area of the vascular arcade corresponding to the bone-spicule hyperpigmentations on fundoscopy. She was otherwise healthy, had above-average intelligence, and normal weight.

Patient 2 is a 57-year-old male who was referred to an ophthalmologist at the age of 51 because of night blindness at the age of 19. His visual acuity at the time of diagnosis was 20/50 in both eyes, and he had constricted visual fields. Fundoscopy at the time showed typical bone spicule hyperpigmentation of the retina, very narrow retinal arteries, and a yellowish macula (Fig. 1b). He was myopic, with a refractive error ODS of -4 D with an astigmatism of −2 D ODS. At the age of 40, he underwent a cataract extraction for posterior subscapular cataract ODS, which had minimal effect on his vision, that had deteriorated to 20/200 ODS. The patient was otherwise healthy, apart from arthropathy of both knees. He had normal intelligence and normal weight. His parents were consanguineous, and one of his four siblings was also diagnosed with RP and otherwise healthy. The patient had two healthy children with his non-consanguineous wife.

### Genotype of patients

In patient 1, exome sequencing yielded biallelic heterozygous variants in *INPP5E*, c.1516C > T, p.(Q506*) and c.847G > A, p.(A283T). As both were novel variants, the pathogenicity of a mild missense variant in *INPP5E*, in combination with a protein-truncating variant, was plausible but lacked conclusive evidence. Similarly, in patient 2, exome sequencing yielded a homozygous variant in *INPP5E*, c.1073C > T, p.(P358L), that was proposed to underlie classic RP previously [33], but only heterozygously in combination with c.1669C > T, p.R557C. Therefore, also for this variant conclusive evidence for the pathogenicity remained elusive.

### Determination of “healthy cilium phenotype”

**Fig. 2:**
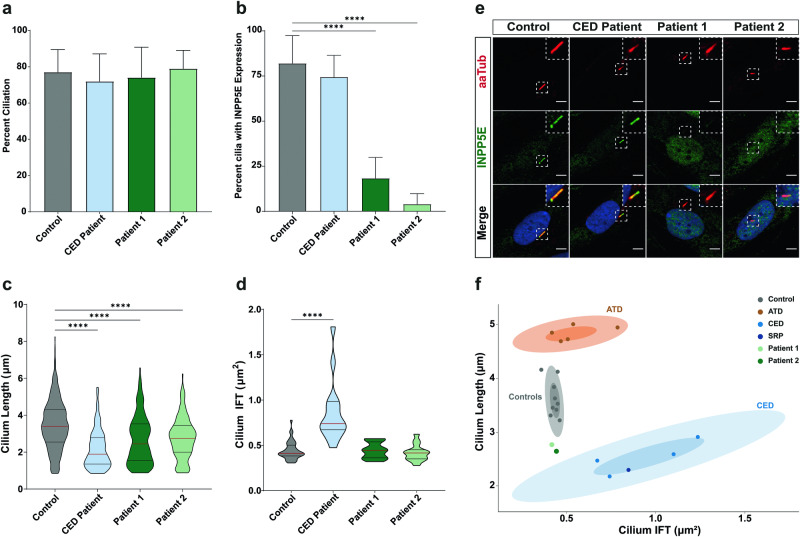
Morphology of the primary cilium is affected in patient-derived fibroblasts. **a** Ciliogenesis of fibroblasts, as a percentage of cells with cilia. There was little variation between control and patient ciliation. Control cells had a ciliation of 77 ± 1.3%, the CED ciliopathy patient 72% ± 2.7%, and the INPP5E patients 74% ± 3.0% and 79% ± 1.8%, respectively. **b** INPP5E localization, as the percent of cilia with INPP5E expression. 82.0 ± 2.1% of control cilia and 74.4 ± 3.1% of CED patient cilia had INPP5E expressed. Both INPP5E patients had a significant loss of INPP5E in the cilia, with 18.3 ± 2.6% and 4.0 ± 1.3% of cilia expressing INPP5E, respectively. **c** Cilium length of control and patient fibroblasts, measured using ALPACA. Both patients showed shorter cilia with a mean of 2.65 ± 0.10 µm and 2.77 ± 0.08 µm when compared to control fibroblasts (3.44 ± 0.06 µm), however, this is more moderate than what is seen in the CED ciliopathy patient (2.16 ± 0.06 µm). **d** Retrograde trafficking was measured using accumulation of IFT88 along the cilium. The mean of control cells was 0.44 ± 0.1 µm^2^, with the INPP5E patients showing no change in accumulation size (0.44 ± 0.1 µm^2^ and 0.42 ± 0.1 µm^2^, respectively). The CED patient showed an increase in accumulation size (0.86 ± 0.3 µm^2^), as observed previously. **e** Representative images of control, CED patient, Patient 1 and Patient 2 fibroblasts stained for α-acetylated tubulin (aaTub, red), INPP5E (green), and DAPI (blue) (Scale bar = 5 μm). **f** Subgrouping of patient and control fibroblasts based on data from Doornbos et al. [30]. Confidence intervals of 0.5 and 0.9 are indicated for each identified cluster—control, CED, and ATD. Patients 1 and 2 from this study group were between the control cluster and CED cluster. (**** denotes *p* < 0.0001) (*N* = 3).

Phenotypic analysis of the cilium was performed by determining ciliation percentage, cilium length, and retrograde IFT trafficking, using parameters previously defined [30]. As a control for these parameters, fibroblast lines from three healthy individuals were used (Supplemental Fig. 1a–c). Comparison of control fibroblasts showed an expected healthy phenotype consisting of a ciliation percent of 77%, a mean cilium length of 3.445 µm, and an IFT88 accumulation measurement of 0.443 µm^2^. Patient-derived fibroblasts from a cranioectodermal dysplasia (CED) skeletal ciliopathy patient (compound heterozygous for *WDR35*; c.A25-3G, p.I9TfsX7 and c.A1877G, p.E626G) were analyzed as an experimental control, as the cell line had previously been shown to exhibit shorter cilia and altered retrograde IFT trafficking when analyzed using ALPACA [30]. These cells showed no difference in ciliation rate (72%), however, a significant decrease in ciliary length (2.157 µm) and an increase in IFT88 accumulation in the cilium (0.833 µm^2^) was observed (Fig. 2a, c, d).

### Sequence variants in INPP5E lead to shorter cilia and disruption of INPP5E ciliary targeting

Next, the two INPP5E patient fibroblasts were analyzed using the same methods. No change in ciliation (Patient 1–74%; Patient 2–79%) or IFT88 trafficking (Patient 1–0.5057 µm^2^; Patient 2–0.4118 µm^2^) was observed in either patient (Fig. 2a, d; Supplemental Fig. 1e, f). However, both patient fibroblasts showed a moderate yet significant decrease in mean cilium length when compared to control cilia, with mean lengths of 2.649 µm and 2.770 µm, respectively (Fig. 2c).

To determine if the patient mutations alter INPP5E within the cells, we evaluated INPP5E localization and expression using IF. Localization of INPP5E was determined by its colocalization with the cilia marker acetylated-α-tubulin. In control fibroblasts INPP5E was present in 82% of total cilia (Supplemental Fig. 1d), and in the CED ciliopathy patient 74% of the total cilia were positive for INPP5E. Strikingly, in both patient cell lines INPP5E localization in the cilium was significantly decreased, with only 18% and 4% of cilia positive for INPP5E, respectively (Fig. 2b).

## Discussion

We identified two patients with progressive retinal degeneration, diagnosed as non-syndromic RP. Through exome sequencing, the patients were found to have novel variants in *INPP5E*, which were believed to be the pathogenic cause of the diagnosed non-syndromic RP, but conclusive evidence remained elusive. Previous studies have identified similar variants to those presented in this paper. Wang et al. [34] reported a patient with heterozygous sequence variants at p.D556E and p.A283V which were predicted to be pathogenic by in silico algorithms, and therefore likely resulted in Leber Congenital Amaurosis in the patient. In our study, patient 1 showed a sequence variant p.(A283T) on one allele at the same amino acid position as identified in this previous patient, though with a different amino acid substitution and in combination with p.(Q506*). Xu et al. [33] reported a patient with non-syndromic RP with compound heterozygous variants at p.R557C and p.P358L that were predicted to be pathogenic by in silico algorithms. In Patient 2 of this study, we identified p.P358L homozygously. As such, both missense variants identified in this study have not been reported previously in the specific substitution of amino acids in the case of patient 1, or as pathogenic variants in the homozygous setting, in the case of patient 2. We therefore set out to acquire this evidence using our previously described “ALPACA” cilium phenotyping tool [30] in combination with the assessment of INPP5E localization using immunofluorescence for specific antibodies.

Molecular phenotyping of patient-derived dermal fibroblasts was performed to determine if the novel variants seen in patients 1 and 2 affect the structure and/or function of the primary cilium. Cells were serum starved for 48 h to induce ciliogenesis, after which it was determined that the variants have no effect on the cells ability to undergo ciliogenesis, nor were there defects in the retrograde trafficking of proteins within the cilium. Previous research has shown that INPP5E interacts with certain IFT proteins, particularly IFT-A proteins responsible for retrograde ciliary axonemal trafficking [23]. This interaction has been identified as a method of regulating sonic hedgehog signaling (SHH) by managing the localization of SHH pathway regulators such as TULP3 and GPR161 [23]. Additionally, previous studies have found that fibroblasts derived from patients with JS have a significantly lower ciliation rate when compared to controls [35]. Taken together, the lack of effect on ciliation and IFT trafficking might provide insight into why the patients in this study have a relatively mild phenotype when compared to patients with JS.

Interestingly, the length of cilia was significantly shorter when compared to healthy controls, though the decrease in length was moderate when compared to the CED patient fibroblast, a more severe ciliopathy related with IFT-A defects [30]. This highlights the importance of INPP5E in regulating ciliary length and stability [14, 25, 36]. The moderate difference in length observed in the dermal fibroblasts, however, may indicate that the overall length of cilia is still sufficient to maintain general cellular homeostasis in other tissues than the retina, as no phenotypic differences in the patients were noted beyond the relatively late onset and slowly progressing retinal degeneration.

To further compare patient phenotypes, the mean cilium length and ciliary IFT measurements of control fibroblasts and patients 1 and 2 were plotted against each other, with the addition of CED, asphyxiating thoracic dysplasia (ATD) and short-rib polydactyly syndrome (SRP) patient fibroblasts – previously analyzed using ALPACA [30] (Fig. 2f). Three clear clusters can be identified in this chart: control fibroblasts, ATD patients with longer cilia than control fibroblasts, and CED and SRP patients with both shorter cilia and higher accumulation of IFT when compared to controls. The two *INPP5E* patients fall between the control cluster and CED cluster, showing a clear separation compared to control however suggesting a milder ciliary phenotype than the more severe CED phenotype. This could be an indication for why the patients present with the milder outcome of non-syndromic RP, however further studies into ciliary phenotyping will help to clarify this.

Finally, α-acetylated tubulin-positive cilia in the two patients showed little to no expression of INPP5E, giving rise to the possibility that the novel variants in these patients lead to a loss of INPP5E in the cilium. It is currently suggested that INPP5E contains four ciliary localization signals (CLS), including two CLSs within the catalytic domain that, due to their close steric proximity, work together to ensure proper localization of INPP5E to the cilium [37]. Though neither patient variant directly alters the proposed CLSs, it is possible that the variants lead to a change in the structure of the catalytic domain thus affecting the two catalytic domain CLSs and leading to a mis-localization of INPP5E. To fully understand the effect of these variants on ciliary localization, though, further studies are required.

**Fig. 3:**
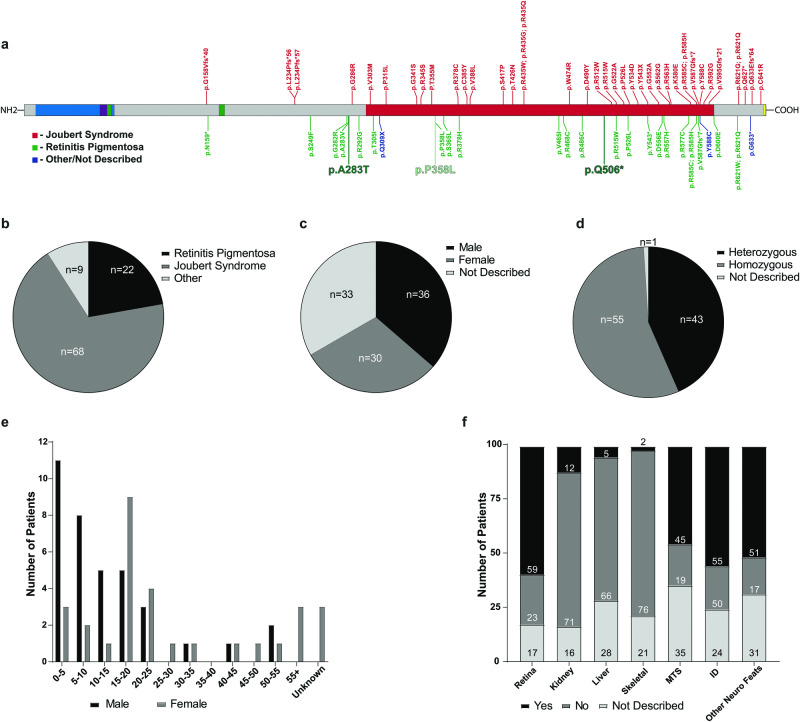
Current knowledge of pathogenic INPP5E variants. **a** Location of variants along INPP5E. The specific colors represent the clinical outcome of each mutation (Red—Joubert Syndrome; Green—Retinitis Pigmentosa; Blue—Other/not described). In-depth descriptions of each known sequence variant can be found in supplemental table 1. Most variants cluster around the phosphatase catalytic domain, however localization of the variant does not determine phenotypic outcome. Comparison of diagnosis (**b**), patient sex (**c**), and type of variant (**d**) as reported in literature. **e** Age and sex comparison of patients. The majority of male patients show symptoms at an early age (0–10 years), while the majority of female patients show symptoms during late teenage years (15–20 years). **f** Prevalence of common ciliopathy phenotypes in patients. Retinal dystrophy, intellectual disability, the molar tooth sign, and other neurological features are most commonly described phenotypes in the cohort.

Pathogenic variants in *INPP5E* are known to lead to a wide range of phenotypes in patients, however, any genotype-phenotype relationship between these variants still remains largely elusive. To clarify if a genotype-phenotype correlation can be determined, a literature search was completed using previously published data of patients with pathogenic variants in *INPP5E*. Within this literature search the age, sex, variant(s), phenotype of patient, and diagnostic outcome were extrapolated and compared (Supplemental Table 1). The majority of these patients (69%) were diagnosed with JS, with non-syndromic RP making up 22% of patients and the remaining were either undiagnosed or had a separate diagnosis, however, no clear correlation could be identified based on either the localization or the type of variant and overall diagnostic outcome in patients (Fig. 3a, b). Additionally, the phenotypes presented by patients varied largely by diagnostic outcome, the most prevalent phenotypes within the cohort were RP (present in 60% of patients), intellectual disability (ID) (present in 56% of patients), an MTS (46% prevalence), and other neurological features, such as ataxia (52% prevalence) (Fig. 3f). Other classical ciliopathy phenotypes, such as kidney, liver, and skeletal anomalies were present, however at a lower rate. Finally, variants do not appear to be sex-dependent (Fig. 3c), however, when looking at age of onset males are more likely to be diagnosed as young children (0–5 years old) while females were more commonly diagnosed as young adults (15–20 years old) (Fig. 3e).

Combined, the findings from previous research highlight the large heterogeneity between patients with variants in *INPP5E* which suggests a more complex mechanism occurring, thus leading to the large variety in phenotypic outcomes seen in patients. While the exact mechanism is still unclear, further studies of patient-derived fibroblasts could help clarify these differences. By performing ciliary phenotyping with ALPACA on both JS and non-syndromic RP patient-derived fibroblasts, we might be able to better understand what complex functions are altered in patients, thus furthering our understanding in the genotype-phenotype relationship of *INPP5E*.

In conclusion, our molecular findings confirm that the novel variants in *INPP5E* are causative for the RP phenotypes seen in the patients. Additionally, this further confirms the possibility to use the ALPACA tool as an assistant in ciliopathy diagnosis and phenotyping.

## Supplementary information

Supplemental Figure 1 - Comparison of controls for all experiments

Supplemental Table 1 - Details of INPP5E patient outcomes from literature

Supplemental References for Supplemental Table 1

## Data Availability

The data generated or analysed during this study can be found within this article and its supplementary files.
