# Supplementary material for: Utilization of automated cilia analysis to characterize novel INPP5E variants in patients with non-syndromic retinitis pigmentosa
Source: Eur J Hum Genet. 2024 May 28;32(11):1412–8. doi: 10.1038/s41431-024-01627-6 (PMC11576733; doi:10.1038/s41431-024-01627-6)
Supplement: Supplementary file 1 — Supplemental Figure 1 - Comparison of controls for all experiments [file 41431_2024_1627_MOESM1_ESM.pdf]

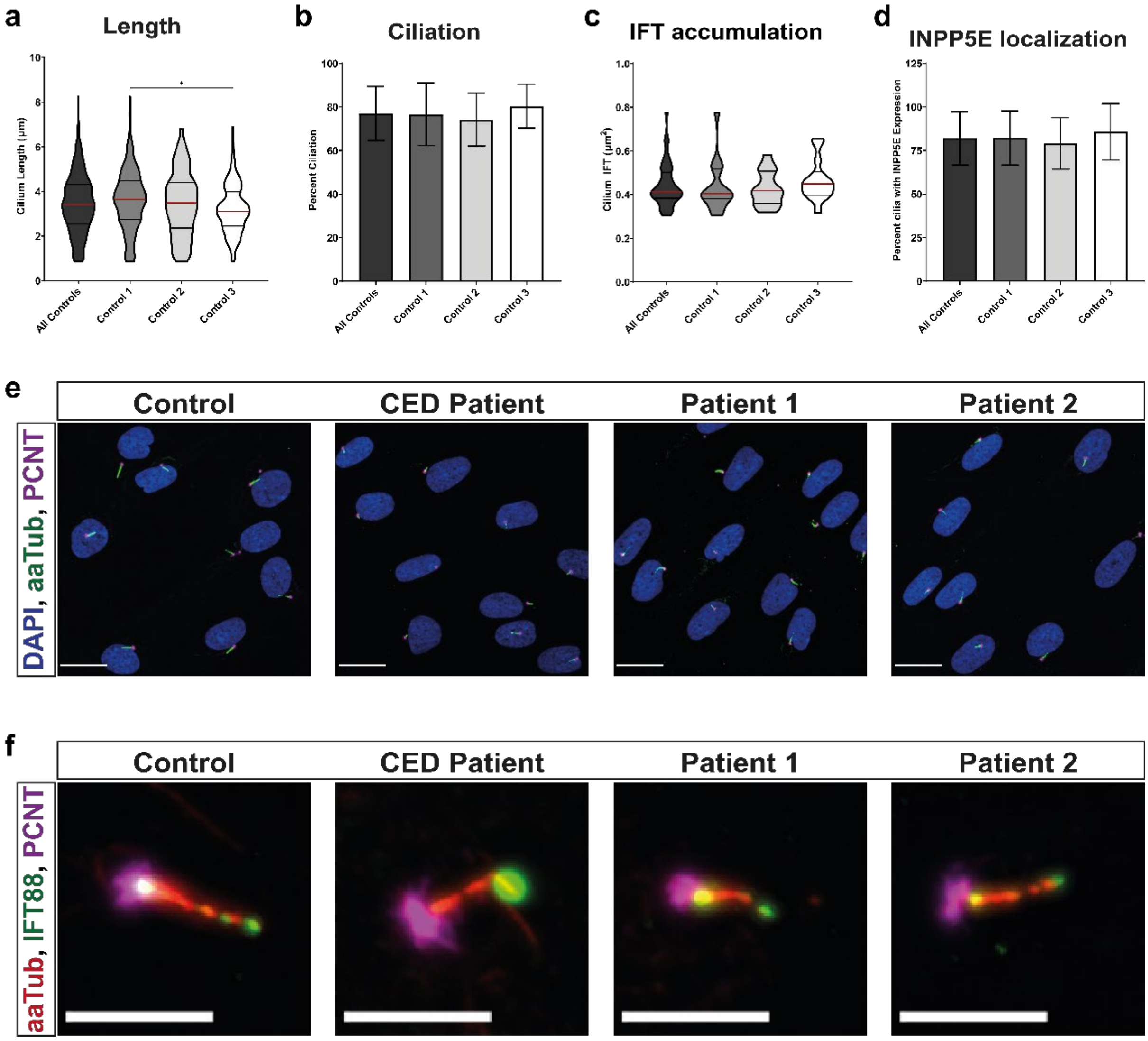

Supplemental Figure 1. Comparison of controls for all experiments. a) Cilium length comparison of control fibroblast lines, measured using ALPACA. Mean cilium length ± SD was compared via a One-way ANOVA analysis (3.62 ± 1.4 µm, 3.4 ± 1.4 µm and 3.2 ± 1.1 µm respectively). **b**) Percent ciliation comparison as the percent of cells with extended axonemes (76 ± 14%, 74 ± 12% and 80 ± 10% respectively). **c**) Retrograde IFT trafficking comparison in controls, measured as the area of IFT88 accumulated along the length of the cilium. Accumulation sizes were 0.44 ± 0.12, 0.43 ± 0.10 and 0.46 ± 0.10 respectively. **d**) Percentage of cilia with INPP5E expression in the axoneme. Controls showed 82 ± 16%, 79 ± 15% and 76 ± 16% of cilia with positive stains for INPP5E (* denotes p=0.0133). **e**) Representative images of percent ciliation in control, CED patient, Patient 1, and Patient 2 stained for DAPI, α-acetylated tubulin (aaTub), and pericentrin (PCNT) (Scale bar = 20µm). **f**) Representative images of IFT88 accumulation measurement stained for aaTub, IFT88, and PCNT (Scale bar = 5µm).
