# Supplementary material for: Utilization of automated cilia analysis to characterize novel INPP5E variants in patients with non-syndromic retinitis pigmentosa
Source: Eur J Hum Genet. 2024 May 28;32(11):1412–8. doi: 10.1038/s41431-024-01627-6 (PMC11576733; doi:10.1038/s41431-024-01627-6)
Supplement: Supplementary file 3 — Supplemental References for Supplemental Table 1 [file 41431_2024_1627_MOESM3_ESM.pdf]

Supplemental References (for Supplemental Table 1)

38. Birtel J, Eisenberger T, Gliem M, Müller PL, Herrmann P, Betz C, et al. Clinical and genetic characteristics of 251 consecutive patients with macular and cone/cone-rod dystrophy. Sci Rep. 2018 Dec 1;8(1).
39. Stone EM, Andorf JL, Whitmore SS, DeLuca AP, Giacalone JC, Streb LM, et al. Clinically Focused Molecular Investigation of 1000 Consecutive Families with Inherited Retinal Disease. Ophthalmology. 2017 Sep 1;124(9):1314–31.
40. Wang H, Wang X, Zou X, Xu S, Li H, Soens ZT, et al. Comprehensive molecular diagnosis of a large Chinese leber congenital amaurosis cohort. Investig Ophthalmol Vis Sci. 2015;56(6):3642–55.
41. Tsurusaki Y, Kobayashi Y, Hisano M, Ito S, Doi H, Nakashima M, et al. The diagnostic utility of exome sequencing in Joubert syndrome and related disorders. J Hum Genet. 2013 Feb;58(2):113–5.
42. Porto FBO, Jones EM, Branch J, Soens ZT, Maia IM, Sena IFG, et al. Molecular Screening of 43 Brazilian Families Diagnosed with Leber Congenital Amaurosis or Early-Onset Severe Retinal Dystrophy. Genes (Basel). 2017 Dec 1;8(12).
43. Sharon D, Ben-Yosef T, Goldenberg-Cohen N, Pras E, Gradstein L, Soudry S, et al. A nationwide genetic analysis of inherited retinal diseases in Israel as assessed by the Israeli inherited retinal disease consortium (IIRDC). Hum Mutat. 2020 Jan 1;41(1):140–9.
44. Brooks BP, Zein WM, Thompson AH, Mokhtarzadeh M, Doherty DA, Parisi M, et al. Joubert Syndrome: Ophthalmological Findings in Correlation with Genotype and Hepatorenal Disease in 99 Patients Prospectively Evaluated at a Single Center. Ophthalmology. 2018 Dec 1;125(12):1937–52.
45. Bachmann-Gagescu R, Dempsey JC, Phelps IG, O'Roak BJ, Knutzen DM, Rue TC, et al. Joubert syndrome: a model for untangling recessive disorders with extreme genetic heterogeneity. J Med Genet. 2015;52(8):514–22.
46. Tiwari A, Bahr A, Bähr L, Fleischhauer J, Zinkernagel MS, Winkler N, et al. Next generation sequencing based identification of disease-associated mutations in Swiss patients with retinal dystrophies. Sci Rep. 2016 Jun 29;6.
47. Gee HY, Otto EA, Hurd TW, Ashraf S, Chaki M, Cluckey A, et al. Whole-exome resequencing distinguishes cystic kidney diseases from phenocopies in renal ciliopathies. Kidney Int. 2014;85(4):880–7.
48. Khan S, Lin S, Harlalka G V., Ullah A, Shah K, Khalid S, et al. BBS5 and INPP5E mutations associated with ciliopathy disorders in families from Pakistan. Ann Hum Genet. 2019 Nov 1;83(6):477–82.
49. Shetty M, Ramdas N, Sahni S, Mullapudi N, Hegde S. A Homozygous Missense Variant in INPP5E Associated with Joubert Syndrome and Related Disorders. Mol Syndromol. 2017 Nov 1;8(6):313–7.
50. Sönmez F, Güzünler-Şen M, Yılmaz D, Cömertpay G, Heise M, Çırak S, et al. Development of end-stage renal disease at a young age in two cases with joubert syndrome. Turk J Pediatr. 2014;56(4):458–61.
51. Travaglini L, Brancati F, Silhavy J, Iannicelli M, Nickerson E, Elkhartoufi N, et al. Phenotypic spectrum and prevalence of INPP5E mutations in Joubert Syndrome and related disorders. Eur J Hum Genet. 2013;21(10):1074–8.
52. Bielas SL, Silhavy JL, Brancati F, Kisseleva M V., Al-Gazali L, Sztriha L, et al. Mutations in INPP5E, encoding inositol polyphosphate-5-phosphatase E, link phosphatidyl inositol signaling to the ciliopathies. Nat Genet. 2009;41(9):1032–6.
53. De Goede C, Yue WW, Yan G, Ariyaratnam S, Chandler KE, Downes L, et al. Role of reverse phenotyping in interpretation of next generation sequencing data and a review of INPP5E related disorders. Eur J Paediatr Neurol. 2016 Mar 1;20(2):286–95.
54. Radha Rama Devi A, Naushad SM, Lingappa L. Clinical and Molecular Diagnosis of Joubert Syndrome and Related Disorders. Pediatr Neurol. 2020 May 1;106:43–9.
55. Wang X, Wang H, Sun V, Tuan HF, Keser V, Wang K, et al. Comprehensive molecular diagnosis of 179 Leber congenital amaurosis and juvenile retinitis pigmentosa patients by targeted next generation sequencing. J Med Genet. 2013;50(10):674.
56. Hardee I, Soldatos A, Davids M, Vilboux T, Toro C, David KL, et al. Defective ciliogenesis in INPP5E-related Joubert syndrome. Am J Med Genet Part A. 2017 Dec 1;173(12):3231–7.
57. Drole Torkar A, Avbelj Stefanija M, Bertok S, Trebušak Podkrajšek K, Debeljak M, Stirn Kranjc B, et al. Novel Insights Into Monogenic Obesity Syndrome Due to INPP5E Gene Variant: A Case Report of a Female Patient. Front Endocrinol (Lausanne). 2021;12(June):1–9.
